# Cognitive and Ocular Factors Jointly Determine Pupil Responses under Equiluminance

**DOI:** 10.1371/journal.pone.0155574

**Published:** 2016-05-18

**Authors:** Tomas Knapen, Jan Willem de Gee, Jan Brascamp, Stijn Nuiten, Sylco Hoppenbrouwers, Jan Theeuwes

**Affiliations:** 1 Department of Cognitive Psychology, Vrije Universiteit Amsterdam, Amsterdam, Netherlands; 2 Dept. of Neurophysiology and Pathophysiology, University Medical Center Hamburg-Eppendorf, Hamburg, Germany; 3 Dept. of Psychology, University of Amsterdam, Amsterdam, Netherlands; 4 Department of Psychology, Michigan State University, Lansing, MI, United States of America; Ghent University, BELGIUM

## Abstract

Changes in pupil diameter can reflect high-level cognitive signals that depend on central neuromodulatory mechanisms. However, brain mechanisms that adjust pupil size are also exquisitely sensitive to changes in luminance and other events that would be considered a nuisance in cognitive experiments recording pupil size. We implemented a simple auditory experiment involving no changes in visual stimulation. Using finite impulse-response fitting we found pupil responses triggered by different types of events. Among these are pupil responses to auditory events and associated surprise: cognitive effects. However, these cognitive responses were overshadowed by pupil responses associated with blinks and eye movements, both inevitable nuisance factors that lead to changes in effective luminance. Of note, these latter pupil responses were not recording artifacts caused by blinks and eye movements, but endogenous pupil responses that occurred in the wake of these events. Furthermore, we identified slow (tonic) changes in pupil size that differentially influenced faster (phasic) pupil responses. Fitting all pupil responses using gamma functions, we provide accurate characterisations of cognitive and non-cognitive response shapes, and quantify each response's dependence on tonic pupil size. These results allow us to create a set of recommendations for pupil size analysis in cognitive neuroscience, which we have implemented in freely available software.

## Introduction

Fluctuations in pupil size are caused by both cognitive and luminance related factors. The recent insight that pupil size reflects brainstem neuromodulatory signals relevant to cognition [1–4] has strongly fuelled interest in the pupil’s responses to cognitive factors [5]. For instance, arousal-dependent modulations of pupil size allow one to track the mechanisms behind the exploration/exploitation trade-off [6–8], and to study decision-making and attentional processes [9] on the timescale of seconds [10–12]. However, fluctuations in the pupil’s size first and foremost regulate the amount of light that falls onto the retina. Most importantly, pupil size tracks luminance variations, with, legend has it, cat pupils being used as luminance measurement devices in early photography. Thus, slight changes in light input, for instance due to blinks and eye movements, may strongly drive the pupil time series. The fact that pupil size fluctuations can have both cognitive and luminance related origins presents us with an inverse problem when measuring only a single number per time unit.

A further complicating factor in the pupillometric approach to cognitive science is that pupil responses to cognitive events are influenced by tonic pupil size [2,10,13], with larger tonic size being associated with smaller phasic (transient) responses. One potential explanation is that arousal level influences both tonic pupil size and event-related cognitive activity [2]. A combined assessment of both cognitive and non-cognitive pupil responses allows us to evaluate this hypothesis, as non-cognitive, reflexive, pupil responses should depend less on arousal state.

Here we aimed to identify distinct contributors to the pupil size time series, and to delineate the optimal methods of its analysis. What duration of event-related responses should be analysed? What is a typical pupil response shape for different types of events? Unlike, for instance, in the field of functional imaging, the methods used to analyse pupil size signals have not yet converged on standard approaches. Our specific aims were threefold: (i) identify separate cognitive and luminance based factors driving the pupil’s time series, (ii) provide response profiles, fitted by gamma distribution functions, of each of the identified components, and (iii) for each component investigate the relationship between phasic and tonic pupil responses. In the process of pursuing these aims, we outline a basic strategy for the analysis of pupil size fluctuations in general, and we provide the tools for this type of analysis in the form of a freely available software package.

To preview our results, we collected pupil data during a simple experiment involving auditory events, and use finite impulse-response (FIR) fitting [14], customary in fMRI, to characterize a well-known arousal-based event response of positive sign, with a characteristic fast impulse-response shape [15]. We also identified several modulations of this established pupil response, to do with uncertainty in the identity and timing of the event. In addition to these cognitive factors, we also identify two strong negative pupillary responses associated with blinks and (micro)saccades. We rule out trivial explanation of these latter responses in terms of recording artifacts, and we argue that these responses, which extend for seconds following the blink or saccade, may be a delayed response to changes in effective luminance. We provide gamma-function descriptions of each of these responses based on a large group dataset (N = 38). We furthermore show that tonic pupil size strongly modulates the arousal-based response, but also that it has a much smaller effect on the luminance-based pupil responses.

**Fig 1:**
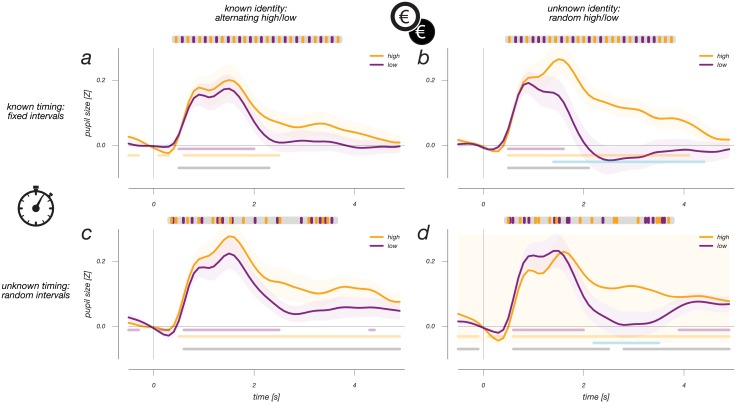
Finite impulse response curves for auditory events in each of the four experimental conditions. Cartoons above show, for each panel, an example time course of the occurrence of the two types of sound over time. All events elicit a transient and fast positive pupil response. Only in the conditions in which the identity of the sound was unpredictable (panels b&d), was there a difference in pupil response between the two sounds. Interestingly, this response difference occurs later in time, relative to the positive event occurrence response that all sounds share. Error regions indicate +/- standard error of the mean in all figures. Horizontal lines indicate, for each of the event types, the periods of significant difference from zero. Cyan lines indicate a significant difference between the two plotted lines, gray lines indicate significant difference between 0 and the mean of the two responses.

We performed an experiment, designed to elicit an established arousal-based pupil response, and to allow verification of the cognitive nature of this response via its modulation by expectancy. Observers looked passively at a blank screen and we recorded pupil size while presenting a stream of brief auditory events. In separate conditions the intervals between tones were either drawn from a narrow gaussian distribution, or from an exponential distribution of equal mean duration. This makes intervals between events either relatively fixed or relatively random, thus varying predictability of event timing [16]. The event stream consisted of two easily distinguishable sounds, designated 'high' and 'low', that signalled different increments to the observer's eventual payment (0.10 € and 0.01 €). These sounds either alternated over time (…-high-low-high-low-…) or were randomly drawn, thereby varying predictability of event identity [17]. Both types of predictability were manipulated independently in a full-factorial 2x2 design (see Fig 1). Prior to event-related analysis, we temporally filtered pupil size time series to produce separate tonic (< 0.02 Hz) and phasic (4 < 0.02 Hz) signal time series. We analysed phasic pupil diameter in an event-related fashion, separately estimating response shapes for both tone types, and also for blinks and eye movements that inevitably occurred.

## Methods

### Participants

The study was approved by the IRB of the Vrije Universiteit Department of Behavioural and Movement Sciences, and conforms to the principles in the declaration of Helsinki. After local IRB approval, 40 participants (including 3 authors, 2 excluded for exceedingly high blink rate) were recruited from the Vrije Universiteit Amsterdam student population. All participants gave written consent and were remunerated both for their participation (9 € per hour) and for each event sound played in the experiment (0.055€ on average). Two versions of the experiment were run (18 and 22 participants, 1 and 2 recording sessions, respectively), in which the sound identity and mean event interval were varied. Varying the sounds that signal value ensures that value responses are not due to the sound’s identity, and the timing of the experiment was adapted to ensure more trials could be recorded from a single participant. Otherwise, the two experimental versions were identical.

### Experimental design

In the fixed timing condition, event intervals were drawn from a Gaussian distribution (mean 3 and 5 s, standard deviation 0.4 and 0.5 s). The slight variation in interval timing precluded metronome-like pacing effects, and diminished the role that anticipation of the exact moment of signals would play in our results while keeping the hazard rate function of event expectation sufficiently peaked in time. Furthermore, this slight randomisation benefits the deconvolution approach in our analysis [14]. In the random timing condition event intervals were drawn from an exponential distribution (mean 1.5 and 3.5 s, adding 1.5 s to each random number drawn to ensure mean interval parity across conditions). Thus, from 1.5 s after the previous sound, event expectation should have been constant over time, as this distribution has a flat hazard rate. In the unknown identity sequences, event identity was drawn pseudorandomly, with a transition probability of 40%. Sequences were played in runs lasting approximately 2–2.5 minutes depending on the intervals drawn for that run, and conditions were presented in pseudorandom order. A total of 96 and 384 events were presented for each condition in the two experimental versions, respectively.

The value signalling sounds were easily distinguishable beeps of 200 ms duration. High value sounds were a 880 Hz tone and a brief cash register sound (version 1 and version 2, respectively), and low value sounds were a 440 Hz tone. We controlled for any residual asymmetric effects due to sound identity by testing the pupil responses in the unknown identity conditions against the known timing, known value condition which employed identical sounds for each participant.

### Pupil recordings and analysis

Pupil diameter of the left eye was recorded at 1kHz using an EyeLink 1000 Tower Mount (SR Research), while participants fixated a black fixation mark (18 min of arc, <0.5 cd/m^2^) on the center of an empty Iiyama 21 inch CRT screen (1024x768, 120Hz, luminance 60 cd/m^2^) placed at 50 cm distance. All experiments and data analysis were performed using custom software written in Python, using the Visionegg and Numpy/Scipy packages.

Blinks were identified based on EyeLink defined standard criteria. These blink events were linearly interpolated from 150 ms before the starting point until 150 ms after the endpoint of the blink. Very rare and brief periods of missing data were likewise interpolated. After interpolation, pupil recordings were band-pass filtered between 0.02 and 4 Hz, using third-order Butterworth filters. Low-pass filtering decreases measurement noise not likely to originate from physiological sources, as the pupil functions as a low-pass filter on fast inputs [15,18]. High-pass filtering removes slow drifts from the signal that are not accounted for by the model in the subsequent deconvolution analysis. Then, data were demeaned per condition, divided by the standard deviation per session (i.e. using the same standard deviation across conditions to avoid contamination of comparisons across conditions) and resampled to 10 Hz. We then analysed these time series using a FIR deconvolution approach, as follows. The analysis estimated time courses in the interval 0.5 s before and 5 s after four event types: two sound types, and blinks and saccades as detected by the EyeLink software. We implemented least-squares deconvolution [14]:

$$h={\left(X^{T}X\right)}^{-1}X^{T}y$$

Here, *y* is the input time series, and *X* is a design matrix consisting of a staggered set of vectors that contain ones at all sample times relative to the event times of which we want to estimate the response, and zeros elsewhere. *h* then contains the concatenated resulting kernels of all the separately defined event types. In our case, *X* had 220 columns (-0.5 to 5 s at 10 Hz, 55 samples per kernel, times two event types, blinks and saccade offsets per deconvolution operation). An explanation of this analysis in code can be found at https://github.com/tknapen/FIRDeconvolution. Non-parametric cluster-based (paired) t-tests [19] were used in all comparisons.

We low-pass filtered all resulting event-related responses (4Hz cutoff), and subtracted a baseline pupil size (0.5–0 s before the event time) from all responses before averaging across subjects. This analysis, which simultaneously estimates pupil responses to sound events and ocular events, ensures that any linear effects of blinks and saccades on pupil dilation are removed from the cognitively interesting time-courses. Since we find that the effect of tonic pupil size is slight for ocular events (although it is significant for the negative response to blinks), this type of linear nuisance regression constitutes a robust cleaning operation on the pupil signal for those interested in cognitive effects.

Estimation of tonic pupil size was done by low-pass filtering the pupil signal using a 3rd order Butterworth filter with a 0.02 Hz cutoff frequency, i.e. gathering the slow components removed from the signals used for deconvolution (disregarding possible cutoff overlap). We then took the values of this low-pass signal, the tonic pupil size, between -0.5 and 0 s relative to event onset, and Z-scored these values on a per-run basis.

Gamma probability density functions and Erlang-gamma functions were fit to pupil response profiles and exponential curves were fit to tonic pupil size evolution within runs, all using the scipy optimize package. The normal Gamma distribution uses the standard shape and scale parameters of the probability density function of the Gamma distribution. In the Erlang version of the function one of the parameters is the t-max, the time to peak of the function. This latter parameter is thus easily interpretable in terms of timing. For the biphasic blink response, we fit a mixture model composed of two gamma functions to the data, the fitted parameters of which we report separately in Tables 1 and 2.

**Table 1:** Parameters of gamma distributions for separate response profiles.

$f\left(x;k;\theta \right)=\frac{k^{-1}e^{-\frac{x}{\theta }}}{\theta^{k}\Gamma \left(k\right)}$	scale (θ)	shape (k)	gain
**Auditory event**	0.314	5.078	0.3
**Blink (negative)**	0.115	8.337	-0.604
**Blink (positive)**	0.178	15.433	0.419
**Saccade**	0.172	6.451	-0.175

**Table 2:** Parameters of erlang-gamma distributions for separate response profiles.

$f\left(x;s;n;x_{max}\right)=\;sx^{n}e^{\frac{-nx}{x_{max}}}$	scale (s)	shape (n)	time to peak (tmax)
**Auditory event**	3.385	4.684	1.448
**Blink (negative)**	-6661.622	7.751	0.844
**Blink (positive)**	0.823	17.061	2.540
**Saccade**	-8.969	3.737	0.917

## Results

**Fig 2:**
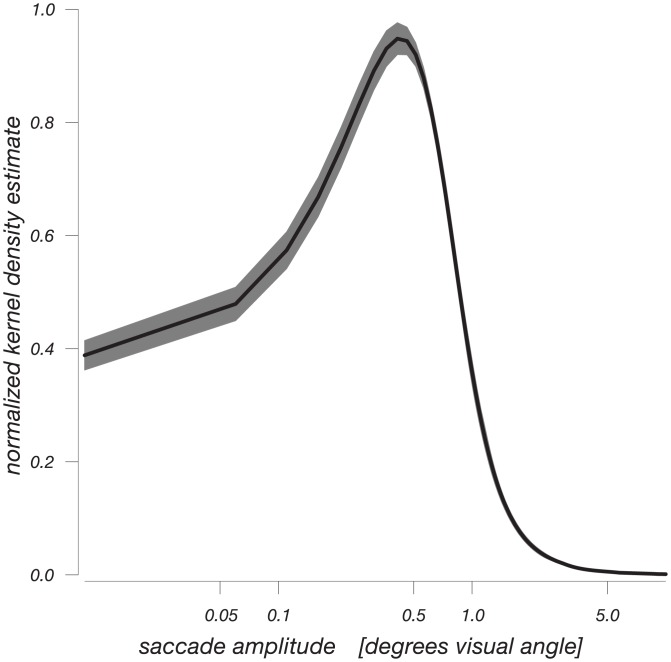
Saccade amplitude distribution. Kernel density estimates of Eyelink-detected saccade amplitude, averaged across conditions. The mode of this distribution falls within one degree of visual angle, a conventional criterium for the classification of microsaccades. Saccade frequency was around 0.8 Hz, and did not differ across conditions.

Tones in all conditions cause a strong phasic pupil dilation (Fig 1) that would typically be interpreted as arousal-related [6,10]. Indeed, the response resembles both the canonical pupil impulse response function [15] and analogous responses in recent literature [6,7,10,13,16]. Fig 1 shows that, in the conditions where tone identity is uncertain, this response differs between tone types, with high-reward tones prompting a more prolonged response (Fig 1b and 1d; cyan horizontal lines indicate moments of significant difference; p<0.05). This further supports an arousal-based account, assuming that unexpectedly high rewards cause stronger (or more prolonged) arousal. Consistent with this idea, the response to reward tones was similarly prolonged in conditions where they occurred at unexpected times (compare Fig 2c and 2d to Fig 2a and 2b; horizontal gray lines specify time-points where the mean of the high- and low-value responses significantly deviated from 0).

**Fig 3:**
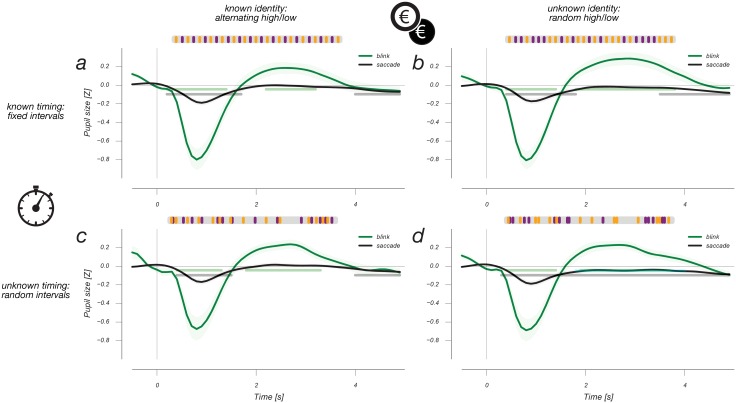
Finite impulse response curves triggered by ocular events. Both blinks and saccades cause an initial negative pupil response, presumably due to the transient increase in retinal illumination after each of these events. The blink response also shows a slower, but positive response. Neither of these two response profiles show any difference between experimental conditions.

We also estimated the response to ocular events during the experiment. The average frequencies of these saccades and blinks were 0.8 Hz and 0.11 Hz, respectively, with no significant differences between conditions. Most saccades had amplitudes below 1 degree of visual angle (Fig 2), meaning that they would conventionally be classified as microsaccades [20]. Fig 3 shows the pupil responses to blinks and (micro-)saccades for all four experimental conditions. There are again no differences between experimental conditions. Blinks cause a large biphasic response in the pupil, first causing a fast transient decrease in pupil size approximately four times the amplitude of both cognitive components described above (*cf*. [12]), followed by a slow increase that lasts up to 5 s.

In other words, the blink-related pupil response far outlasts the blink itself, ruling out explanations in terms of recording artifacts immediately surrounding the blink itself. For saccades we find a somewhat faster change in pupil size marked by a decrease followed by a recovery. This is a novel finding, to our knowledge. As in the case of blink-related pupil responses, these saccade-induced pupil responses are not likely to be due to recording artifacts, such as foreshortening of the pupil’s image in the video-based eye tracker. Foreshortening would approximately follow the cosine of the change in gaze angle resulting from the saccade, and as the average saccade amplitude is <1 degree of visual angle (see Fig 2) this would amount to minute changes in measured pupil size. Specifically, rotating the eye by an angle of 1 degree affects pupil size by about 1% [21–23], whereas the pupil size changes we report are an order of magnitude greater. Indeed, earlier reports of these foreshortening effects intentionally diverted gaze along larger parts of the screen [21–23], which may amount to dozens of degrees of visual angle. Furthermore, whereas altered gaze angle is the immediate effect of a saccade, the time course of the saccade-induced pupil size fluctuations is not instantaneous but develops across several seconds, similar to luminance-induced pupil size fluctuations [18,24]. In sum, accounts of these ocular pupil responses in terms of recording artifacts would not explain either their timecourse and their magnitude, and we prefer an account in terms of brief changes in effective luminance that accompany ocular events (see Discussion section). In light of the slow timecourse of the pupil responses to these ocular events, simple removal of saccade or blink events themselves from a recorded pupil timecourse would not adequately counteract their influence on the pupil signal. In contrast, the present approach of simultaneously regressing these events’ responses along with responses of interest, removes any linear influence of these events’ responses.

**Fig 4:**
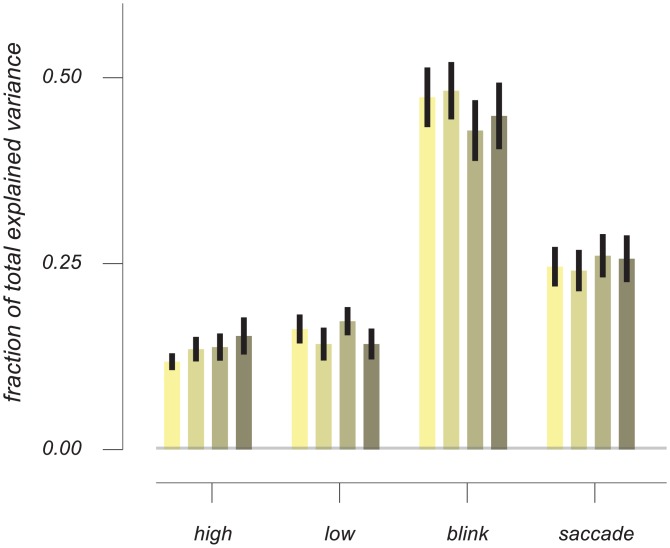
Fraction of total explained variance for each of the response profiles. Ocular event response profiles together explain almost four times more variance in the signal than auditory events combined. Colours represent the different conditions in the following order; 1. known timing, known identity, 2. known timing, unknown identity, 3. unknown timing, known identity, 4. unknown timing, unknown identity. Error bars are +/- 1 standard error of the mean across subjects.

How important are these responses, caused by ocular events, in the composition of the full pupil signal? To quantify this, we calculated the contributions of each of the four event types (two tones, saccades and blinks) relative to the total amount of variance explained in the FIR analysis. Strikingly, the resulting fractions were consistently larger for the ocular events (jointly accounting for around 70% of total explained variance, see Fig 4) than for the events that were manipulated experimentally. These results highlight the importance of including these ocular events in one’s analysis, especially because their occurrence can correlate with cognitive experimental manipulations [20], and traits [25]. Within the 70% variance explained by ocular events, the contribution of blink-related responses is markedly larger than that of saccade-related responses. Note, however, that this is unrelated to any transient change in recorded pupil size due to the eyelid occluding the pupil during a blink (this segment of the recorded timecourse was not included; see methods). Instead, both the blink-related responses and the saccade-related responses are seconds-long physiologically-driven changes in pupil size that follow the actual ocular events.

**Fig 5:**
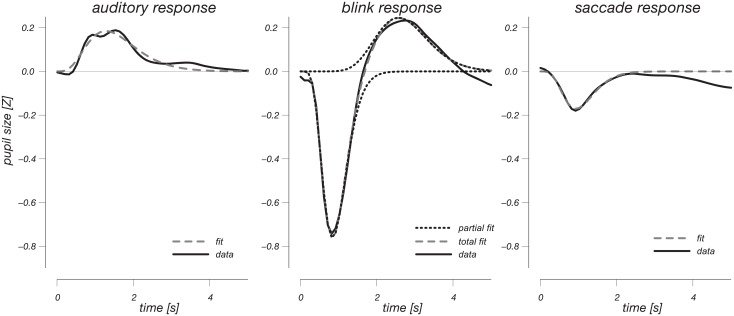
Response curves fit to gamma distribution probability density functions. The same scale is used for all curves. The data, averaged across 4 conditions for event, blink and saccade response profile panels, are plotted in solid black lines, whereas the best-fitting curve is plotted using dashed grey lines. The dotted lines in the blink panel show the two gamma shapes that together define the full biphasic shape, listed separately in Tables 1 & 2.

Next we performed fits of these observed response profiles using (double) gamma distribution probability density functions, to quantitatively describe the identified response profiles according to two equivalent formulations (Gamma and Erlang-Gamma equations, see Tables 1 and 2). These response profiles can in future work be used in general linear model-type regression analyses on pupil size data, analogous to the approach which has become standard in fMRI research [26]. The relatively large number of subjects (38) provides a clear average response shape that is well fit using these functional descriptions. Fig 5 and Table 1 show response profiles fitted by gamma distributions (double gamma for blinks), and the best-fitting parameters of the distributions, respectively. Keeping the amplitude axis identical across panels clearly illustrates how strong the blink response is, relative to the other response profiles.

**Fig 6:**
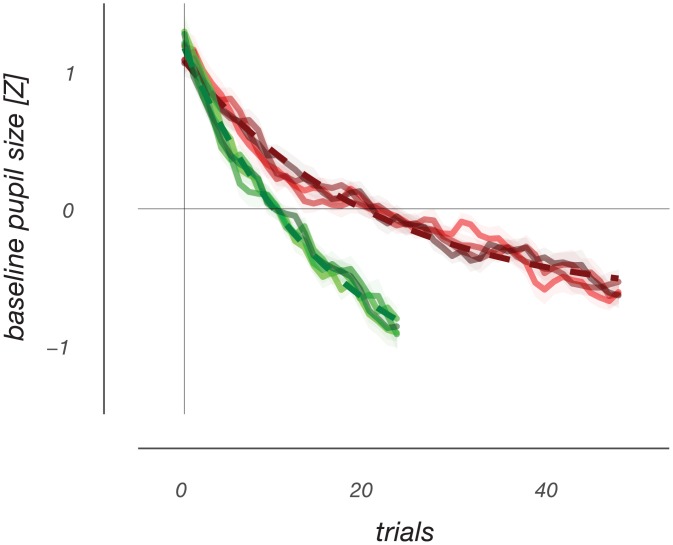
Slow exponential decrease of tonic pupil size over time. During a single run, tonic pupil size starts high and falls over the course of the run. Two experimental versions had different amounts of trials per recording run and are plotted in different colors. For the red line, trials had an average duration of 3 s, whereas for the green line the average trial duration was 5 s. The different lines per experimental version depict the tonic pupil timecourse for the different experimental conditions, ordered as in Fig 6, and show no difference between conditions. The dashed lines are best- fitting exponential curves. When accounting for the difference in average trial duration, the time constants of these fits are roughly similar, with a slight ~20% difference.

**Fig 7:**
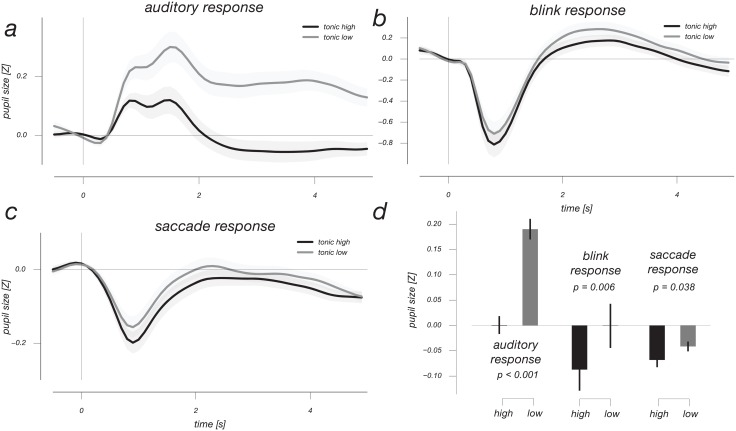
Transient pupil responses are differentially sensitive to tonic pupil size. Events of each type were characterised based on above-median or below-median tonic pupil size. Data were averaged across 4 conditions for auditory, blink and saccade panels a,b,c. The response amplitude and shape of auditory responses changes drastically as a function of tonic pupil size. This effect is evidenced by the bar charts (panel d), showing average responses throughout periods in which the response profiles in question were significant in Figs 1 and 3. For each condition there is a significant scaling due to tonic pupil size, this effect is by far the largest for the ‘mean’ response profile, related to the canonical arousal-mediated Response profile.

One important finding in recent pupil work has been the fact that large tonic pupil size is associated with smaller phasic pupil responses [2,10,13]. Were there noteworthy variations in tonic pupil size in our experiment, and is our characterization of the different response profile shapes affected by tonic pupil size? Indeed, aside from non-systematic variations in tonic pupil size, we found a gradual decrease over the course of individual runs in our experiment (Fig 6). This decrease was well fit using an exponentially decaying function, and showed similar timescales for both versions of our experiment in which inter-trial timing was different (see methods). Moreover, when we re-computed transient response profiles after a median-split based on tonic pupil size a considerable influence of tonic size on response profiles was apparent (Fig 7). Of note, this influence of tonic pupil size on more transient response profiles is most pronounced for the cognitively interesting responses to auditory events. Interestingly, these pupil responses to auditory events change not only in terms of their amplitude, but also their shape as a function of tonic pupil size.

## Discussion

We identified several cognitive and ocular factors that influence pupil size. We find a classical arousal-based pupil response [6,7,10,15,16], which is modulated by the predictability of event timing, and becomes more sustained for unexpectedly favorable events. Thus, this response seems to track the observer’s surprise associated with an event [27]. Importantly, in spite of strict instructions to maintain fixation and blink as little as possible we find that the responses to ocular events contribute more variance to the pupil size signal than responses to our experimentally manipulated events. Furthermore, we find that tonic pupil size influences these latter responses to an extent that can be highly relevant in cognitive experiments. Having identified these factors, below we present specific recommendations to help minimize the influence of non-intended effects on pupil size in an experimental setting, and we will also link to our freely available software package that can help implement these recommendations. First however, we will provide tentative interpretations of our empirical data.

The pupil’s responses to blinks and saccades share a fast negative, or myotic, component with a similar time-course. We interpret this shared myotic response as a reaction to the post-blink and post-saccadic increase in retinal illumination. In the case of blinks, this is the cessation of darkness during the blink (a large luminance transient), and in the case of post-saccadic myosis this could be the novel illumination of parts of the retina that were previously not exposed to the display’s image (a relatively small luminance transient). The relative strength of these luminance transients is a possible cause of the difference in amplitude between the blink and saccade responses. The myotic responses to increases in incoming light are known to be faster than the opposite dilatory, or mydriatic, response to decreases in incoming light [28,29]. This large difference in timescale may explain the slow positive component of the blink response, possibly due to the lack of retinal illumination during the blink. Of course, it is standard practice to interpolate blinks, and/or exclude the data in the period following blinks from one’s analysis. However, our present findings indicate that we should exclude approximately 5 seconds of data after each blink to ensure that results are not tainted by blink-induced effects. This is a longer period than previously implicated [12], and accounts for a significant portion of all data given a blink rate of 0.11 Hz on average in our experiment. If our interpretation of the identified saccade-triggered responses as luminance-mediated constrictions is correct, their sign and amplitude should depend on the endpoint of the eye movement as the fovea most strongly drives the pupil’s constriction [30]. Although we could not analyse this in our dataset because of the diminutive size of the fixation mark, we emphasise that this possible influence of saccade endpoint luminance could easily be corrected for in the analysis recommendations we propose below.

High tonic neuromodulator release can cause large tonic pupil size and promote explorative over exploitative decision-making [2,7]. We find that tonic pupil size follows a stereotypical pattern during the first three minutes of a participant-initiated recording. This may reflect an explorative decision on the part of the participant at the start of the recording, after which tonic pupil size descends slowly into the exploitative domain. We find similar time-courses for runs with different inter-trial intervals, but the timescales of these slow processes may well be flexible, and depend on specific task demands. We believe that the investigation into the dynamics of the tonic-phasic pupil size interaction will in the future shed light on the functional mechanisms of neuromodulatory action in the brain.

The scaling and deformation of transient pupil responses as a function of tonic pupil size indicates non-stationarity in pupil size timeseries, very similar to non-stationarities observed in fMRI responses [31]. This is not an effect remedied by the often-used method of per-trial baseline subtraction, which would do nothing to compensate the effect of tonic pupil size on the amplitude and duration of transient responses. Indeed, the influence of tonic pupil size is clear in our plots that align all pupil baselines to 0 (Fig 7). Interestingly, we find that low tonic pupil size is associated with upward response profile changes for both positive and negative pupil responses, indicating that low tonic pupil size does not simply increase transient pupil response gain. We did not record behavior in our experiment, but it would be interesting to see whether choice behavior and tonic pupil size follow a similar evolution during the initial period of a recording [7,8,32].

From these findings, some strong recommendations for the analysis of pupil size in cognitive science can be distilled. First, a promising avenue of analysis is the possibility to use time-course regression analyses similar to the analysis methods presently employed. Previous work [33–35] has used regression analysis to find pupillary responses correlated with distinct model-based variables. Based on the finding that event-related responses are influenced by tonic pupil size, we advocate that analyses focused on phasic event responses use the tonic pupil size at each event as a covariate. This approach allows analysis of the very interesting tonic-phasic pupil size interaction, and helps provide a better estimate of phasic responses. Furthermore, the structured evolution of tonic pupil size during a recording suggests either using temporally balanced designs or excluding the first three minutes of data, and paying particular care that findings regarding phasic responses are not simply inherited from the tonic baseline. The strong influence of blinks and saccades on the pupil time course is a compelling reason to add these events to one’s analysis as nuisance events, again making regression analysis an attractive option.

We have developed a package of Python code and have made it available on GitHub as a repository called FIRDeconvolution [36]. This package allows convenient implementation of FIR analyses. To promote usability, the repository includes a tutorial that demonstrates the package's basic functioning. Furthermore, we have included an ipython notebook that implements a specific pupil size preprocessing strategy based on our findings and recommendations. Specifically, it performs linear blink interpolation, temporal band-pass filtering, after which blink- and saccade-related contributions to the pupil size time series are estimated with a GLM analysis using blink and saccade kernels such as described in Tables 1 & 2. We note that if saccades are made to areas of higher or lower luminance, separate regressors could be used depending on the saccade endpoint luminance (as FIRDeconvolution allows for, too). The residuals of this GLM are then used for further analysis, potentially after adding tonic pupil size to these band-pass filtered residuals to reinstate the broadband pupil size time series.

We foresee that following the above recommendations, and potentially using the code provided, will enhance both the sensitivity and the accuracy of analyses, and thereby bolster the utility of pupillometry for the study of cognitive processes.
